# Association between Depressive Symptoms and Cognitive Function in Persons with Diabetes Mellitus: A Systematic Review

**DOI:** 10.1371/journal.pone.0160809

**Published:** 2016-08-15

**Authors:** Sofia M. Danna, Eva Graham, Rachel J. Burns, Sonya S. Deschênes, Norbert Schmitz

**Affiliations:** 1 Department of Epidemiology, Biostatistics and Occupational Health, McGill University, Montreal, Quebec, Canada; 2 Douglas Mental Health University Institute, Montreal, Quebec, Canada; 3 Department of Psychiatry, McGill University, Montreal, Quebec, Canada; 4 Montreal Diabetes Research Centre, Montreal, Quebec, Canada; Nathan S Kline Institute, UNITED STATES

## Abstract

Depression and diabetes are independent risk factors for one another, and both are associated with increased risk of cognitive decline. Diabetes patients with lower cognitive function are more likely to suffer poorer health outcomes. However, the role of depression in cognitive decline among people with diabetes is not well understood. This systematic review assessed whether adults with comorbid diabetes and depression or depressive symptoms exhibit greater cognitive decline relative to individuals with diabetes alone. Searches were run in CINAHL, the Cochrane Central Register of Controlled Trials, EMBASE, PsycINFO, and PubMed (MEDLINE) with no time or language restrictions. Studies were eligible for inclusion if they were of any quantitative study design, included participants aged 18 years or older with diabetes mellitus of which some must have presented with current depression, and measured cognition as an outcome. The Cochrane Collaboration’s Risk Of Bias In Non-randomized Studies–of Interventions tool was used for quality assessment of each study and its collected outcome. Fifteen articles were included in the final analysis. The high degree of heterogeneity in exposures, outcomes, and participant characteristics precluded a meta-analysis of any of the studies, and the risk of bias observed in these studies limits the strength of the evidence. Nonetheless, this review found the presence of comorbid depression was associated with poorer cognitive outcomes than for persons with diabetes alone. While large-scale preventive efforts must address epidemic levels of diabetes and its comorbidities, on the patient level healthcare professionals must be cognizant of the added difficulties that depression poses to patients and the extra support required to management diabetes in these cases. This systematic review is registered with the University of York Centre for Reviews and Dissemination under registration number 2015:CRD42015025122.

## Introduction

### Rationale

Diabetes mellitus is a chronic metabolic condition characterized by insufficient production of insulin or inability to use the insulin that the body produces, resulting in problems regulating blood sugar. [1] According to the World Health Organization, it is estimated that 422 million people had diabetes in 2014, representing 8.5% of adults worldwide. [2] The prevalence of diabetes has been increasing consistently for three decades, and is projected to continue rising. [2] Diabetes is associated with several complications including loss of vision, kidney failure, cardiovascular disease, and lower limb amputation [2]; and recent evidence indicates that it may lead to increased cognitive decline. A meta-analysis published in 2014 of 25 studies found small but significant deficits in a wide range of cognitive domains for persons with diabetes relative to those without diabetes (Cohen’s *d* = -0.25). [3] This increase in risk was also found for dementia: an earlier meta-analysis of 15 prospective population-based studies showed the presence of diabetes in older adults was associated with 47% increased risk of all dementia, 39% increased risk of Alzheimer’s Disease, and 138% increased risk of vascular dementia relative to the absence of diabetes. [4] For the purposes of this review, the use of the term “diabetes” will refer to all or unspecified types of diabetes unless otherwise noted.

Depression is a common mental illness, with an estimated 350 million people affected around the world, [5] that is similarly associated with cognitive decline as measured by neuropsychological assessments and rates of dementia. A 2001 meta-analysis showed clear associations between depression and subsequent dementia and Alzheimer’s Disease: a 101% increase in relative risk was found among seven case-control studies, and an 87% increase in relative risk was found among six prospective studies. [6] A later meta-analysis of nine case-control studies and 11 cohort studies indicated similar results: relative to absence of depression, depression was associated with a 103% increase in odds of dementia in case-control studies and 90% increase in odds in cohort studies. [7] The most recent meta-analysis to date included 24 studies and assessed executive function, memory, attention, and reaction time. Similarly, it showed persons with current and remitted depression had significant moderate deficits compared to persons without depression. [8]

In addition to being risk factors for cognitive decline, studies show that diabetes and depression are independent risk factors for each other. Two meta-analyses assessing depression as a risk factor for diabetes found approximately 38% increase in risk of incident diabetes in people with depression relative to people without depression. [9, 10] A third meta-analysis showed a greater association of 60% increase in risk of onset of type 2 diabetes. [11] Two meta-analyses considering diabetes as a risk factor for depression found and diabetes was associated with a 25% increase in risk of incident depressive symptoms, [12] and that type 2 diabetes was associated with a 15% increase of depression. [11] As a result of this bidirectional relationship, comorbid depression is common in people with diabetes, with estimates ranging between 10.6% and 25.3%. [13–17]

The presence of depression in people with diabetes is associated with problems in diabetes management and several health outcomes. Evidence shows comorbid depression in diabetes is associated with lower adherence to self-care behaviours such as diet, physical activity, use of medication, and glucose monitoring, [18, 19] as well as poor glycemic control, [20] and microvascular and macrovascular complications. [21–23] Comorbid depression in diabetes also represents greater financial burden, as it is associated with higher healthcare costs relative to diabetes only. [24] Given this tendency toward poorer outcomes, it is likely that the simultaneous presence of both depression and diabetes similarly be associated with worse cognitive functioning than is seen in people with diabetes alone. However, few studies have explicitly quantified this comparison, and no synthesis of the evidence is available to date.

### Objectives

The objective of this review is to synthesize and critically analyze information from studies of any methodological design that compare cognitive outcomes of people with diabetes and comorbid depression to those with diabetes and no comorbid depression. Only cases of current depression are included in order to ensure that depression or depressive symptoms are active exposures in a relevant timeframe to the cognitive outcomes being measured in each study. For the purposes of this study, depression or depressive symptoms occurring in the two preceding years was considered current. It is possible that lifetime exposure to depression is associated with subsequent cognitive decline, but due to the wide variation this exposure may take (e.g., single episode five decades prior, highly recurrent episodes throughout the life course), this review seeks to observe the association between cognition and immediately comorbid depression. In reviewing the existing literature we aim to determine whether individuals with comorbid depression exhibit an increased risk for cognitive decline relative to those with diabetes alone.

## Methods

Prior to performing the review, a protocol was registered with the University of York Centre for Reviews and Dissemination under registration number 2015:CRD42015025122. [25]

### Search

A comprehensive search strategy was developed to capture articles reporting on depression or depressive symptoms, diabetes, and a wide range of cognitive domains and outcomes. A basic search strategy was developed in PubMed and later adapted to other databases (Table 1).

**Table 1:** Search strategy.

Diabetes	Depression	Cognitive outcomes
diabet*[Text Word]	antidepress*[Text Word]	alzheimer*[Text Word]
Diabetes Mellitus[MeSH Terms]	depressant*[Text Word]	amnes*[Text Word]
elevated blood glucose[Text Word]	depressed[Text Word]	cogniti*[Text Word]
elevated blood sugar[Text Word]	depressi*[Text Word]	Cognition[MeSH Terms]
high blood glucose[Text Word]	Depression[MeSH Terms]	Delirium, Dementia, Amnestic, Cognitive Disorders[MeSH Terms]
high blood sugar[Text Word]	Depressive Disorder[MeSH Terms]	dementia[Text Word]
hyperglycemi*[Text Word]		executive function*[Text Word]
Hyperglycemia[MeSH Terms]		Executive Function[MeSH Terms]
insulin resistan*[Text Word]		Learning[MeSH Terms]
Insulin Resistance[MeSH Terms]		learning[Text Word]
		MCI[Text Word]
		memory[Text Word]
		mental process*[Text Word]
		mental status[Text Word]
		mini mental state[Text Word]
		MMSE[Text Word]
		processing speed[Text Word]
		reasoning[Text Word]
		verbal fluency[Text Word]

Searches were run on August 6, 2015 in CINAHL, Cochrane Central Register of Controlled Trials (CENTRAL), EMBASE, PsycINFO, PubMed (MEDLINE) without time or language restrictions. Studies of any quantitative search design were accepted, including clinical trials or observational studies, whether case-control, cohort, cross-sectional, or longitudinal. Studies were included if participants were aged 18 years or older and had diabetes mellitus of any type, and if some participants had current depression or depressive symptoms in addition to diabetes. Current depression was defined as meeting depressive criteria at baseline or within two years prior to baseline. While this did not exclude people who had experienced depression or depressive symptoms more than two years before the study, it only retained people who had experienced them in this more relevant timeframe. Each study must have measured a cognitive outcome, meaning any measure of any cognitive domain (e.g., executive function, processing speed) or general cognitive functioning or deterioration (e.g., mild cognitive impairment, dementia). All studies must have allowed for a comparison of cognitive functioning between the depression groups. Studies were not excluded on the basis of population characteristics, study setting, publication status, or any other methodological criteria.

### Study selection

Search hits were imported into EndNote and deduplicated. Two reviewers (SMD and EG) independently screened the titles and abstracts of all unique hits for eligibility and resolved disagreements by consensus. The full texts of the selected studies were then screened for eligibility and disagreements again resolved by consensus. The reason for excluding an article during the full-text screening was recorded.

### Data collection

The two reviewers independently collected data using a pilot-tested extraction form and resolved disagreements by consensus. Data extracted included participant characteristics (number of participants with diabetes and comorbid depression, recruitment/sampling information, age, sex, ethnicity, educational attainment, socio-economic status), study characteristics (country, setting, study design (including data collection points and total follow-up), informed consent), diagnostic criteria used to determine diabetes status (including diabetes type), depression status (including timeframe of measurement), and results about cognitive outcomes. Results extracted included the most pertinent analysis conducted (model specification, statistical and design-based adjustment), effect measure reported, as well as risk of bias for each study. If information on a result of interest was not reported, the lead author was contacted by email, with up to two follow-up emails if no reply was received.

### Quality assessment

The methodological quality of the each study and its most relevant study result was assessed using the Risk Of Bias In Non-randomized Studies–of Interventions tool (ROBINS-I, formerly called A Cochrane Risk of Bias Assessment Tool: for Non-Randomized Studies of Interventions) [26] during the process of data extraction. ROBINS-I provides a detailed framework for assessment and judgement of risk of bias that may arise due to confounding, selection of participants into the study, measurement of interventions, departures from intended interventions, missing data, measurement of outcomes, and selection of reported results. The ROBINS-I tool is equally appropriate for cross-sectional and longitudinal non-randomized studies as quality assessments are independent of study design. Based on review of the literature concerning confounders, age, ethnicity or race, physical activity, and education were identified as critical confounders that required suitable adjustment for study results to have low risk of bias. Each domain is determined to exhibit low, moderate, serious, or critical risk of bias. Low risk indicates that the study is “comparable to a well-performed randomized trial” in the domain being evaluated. Moderate risk of bias indicates the study is “sound for a non-randomized study” but not comparable to a rigorous randomized trial. Serious risk of bias indicates the presence of “important problems,” while critical risk of bias indicates the study is “too problematic … to provide any useful evidence on the effects of intervention”. If insufficient information is provided to determine the risk of bias of a certain domain, the domain is marked as having no information. The overall risk of bias of each study was equal to the most severe level of bias found of any domain. [26] All studies were analyzed using this tool regardless of whether the original study design included randomization to other exposures, thus ensuring that risk of bias was assessed specifically for the comparisons of interest to this review. The results of the review were grouped according to the methodological quality of each study’s selected outcome.

It is important to note that the quality assessment reflects how well a specific result evaluated the association of interest to this review, regardless of the objectives of the original study. The study outcome of interest compared cognitive outcomes among depression groups with the smallest degree of bias, but was often not the main result of the study and did not match the study’s substantive focus. As a result, these quality assessments only apply to this review and not to the methodological quality of the study for its intended objectives.

Reporting of the systematic review followed PRISMA guidelines (S2 File). [27]

## Results

### Study selection

**Fig 1:**
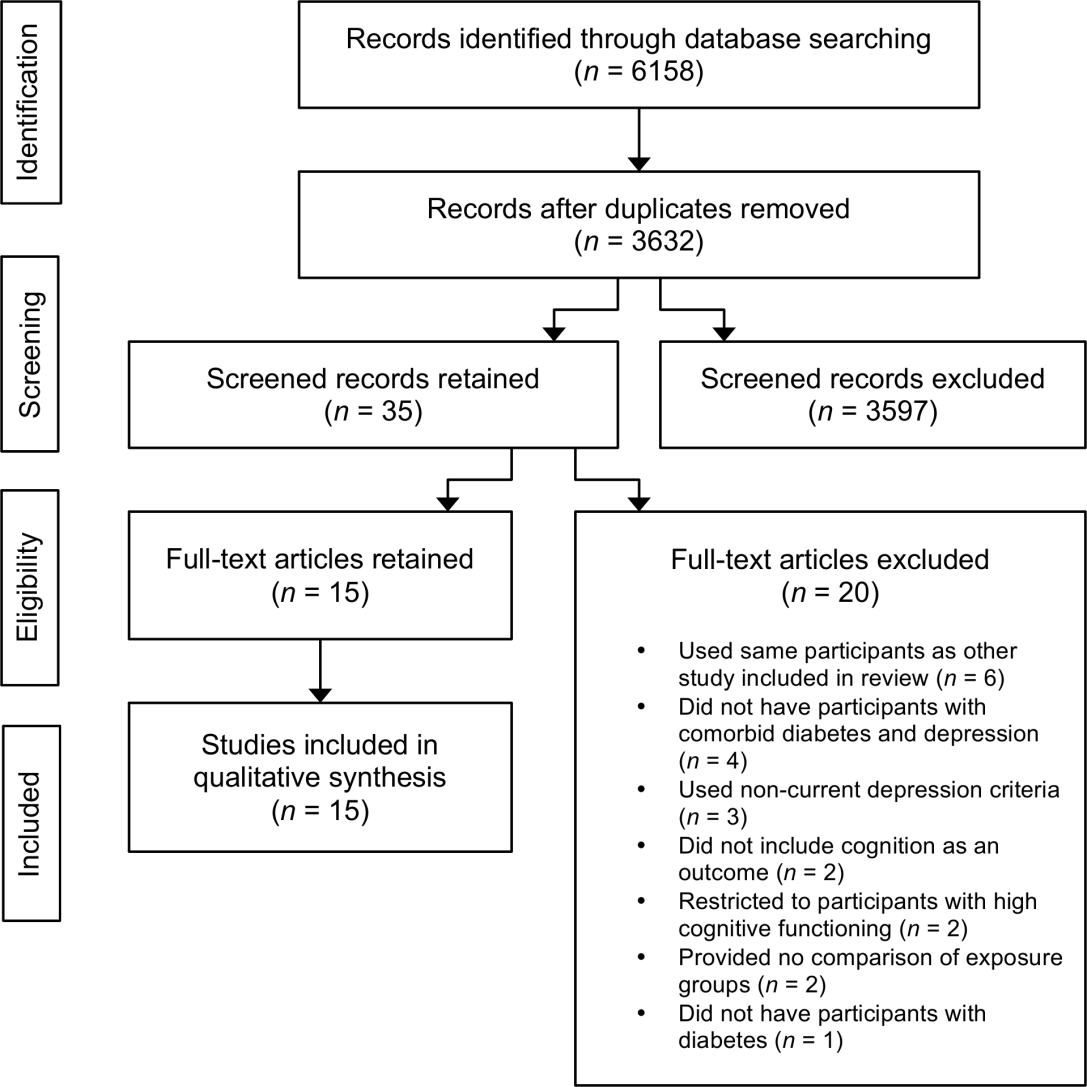
PRISMA Flow Diagram.

Three thousand six hundred and thirty-two (*n* = 3,632) unique articles were retrieved from searching five major databases, of which 35 were found to be relevant in the title and abstract screening. Twenty articles were eliminated in the full-text screening for not having met the inclusion criteria (Fig 1, S1 File): six studies included the same sample participants as other studies in the review, four did not have participants with comorbid depression and diabetes, three used non-current depression criteria, two measured cognition as an exposure and not an outcome, two restricted to participants with high cognitive functioning only, two did not provide a comparison of the exposure groups, and one did not have participants with diabetes. In cases of studies with repeated sample participants, all studies and their results were assessed for quality and the one with lowest risk of bias was selected. Fifteen studies were retained for final analysis. [28–42]

### Results by risk of bias

**Fig 2:**
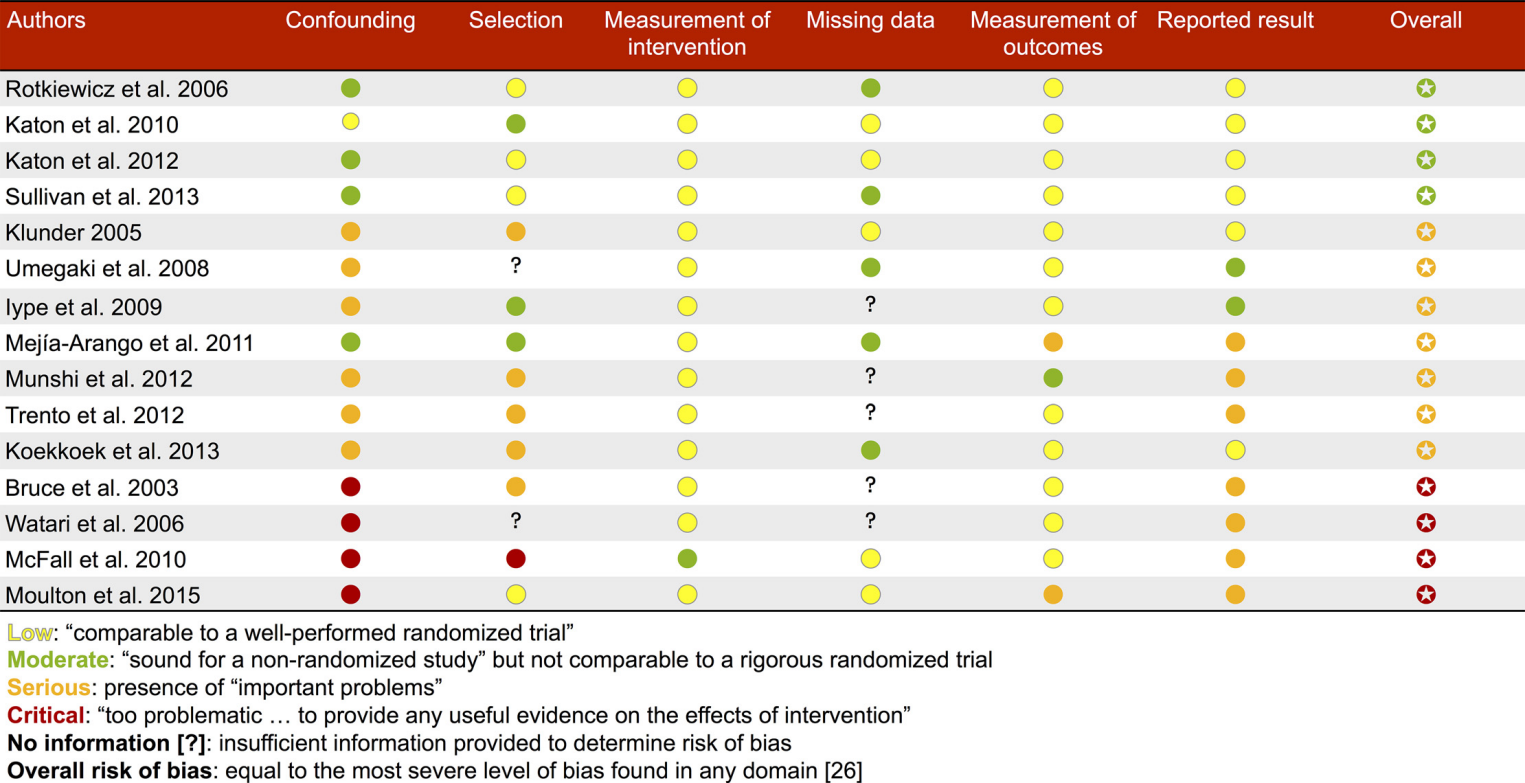
ROBINS-I risk of bias assessment.

The main findings are discussed below, organized by overall risk of bias of the studies. Key study characteristics are summarized in Table 2, and details of the risk of bias assessments for each study are reported in Fig 2. It should be noted that the ROBINS-I domain regarding departures from interventions was omitted as it did not apply to this review, as the exposure of interest was depression at baseline, regardless of whether it persisted or not. As stated previously, the overall risk of bias of each study was equal to the most severe level of bias found of any domain.

**Table 2:** Study characteristics.

Authors	Year	Sample size	Country	Age	Sex (% female)	Study design (follow-up)	Diabetes type and criteria	Depression criteria	Cognitive criteria
Bruce et al. [28]	2003	223	Australia	70+	49.8	Cross-sectional	Types 1, 2; criteria not stated	Even Briefer Assessment for Depression	Mini Mental State Examination
Klunder [29]	2005	73	USA	60–85	43.8	Cross-sectional	Type 2; doctor diagnosis, fasting plasma glucose, HbA1c level	Beck Depression Inventory	California Verbal Learning Test
Rotkiewicz et al. [30]	2006	808	USA	65+	60.0	Prospective cohort (7 years)	Type not specified; self-report	Center for Epidemiologic Studies–Depression	Mini Mental State Examination
Watari et al. [31]	2006	40	USA	30–80	70.0	Cross-sectional	Type 2; doctor diagnosis	DSM-IV criteria for major depression	Mini Mental State Examination
Umegaki et al. [32]	2008	907	Japan	65+	54.5	Cross-sectional	Type not specified; HbA1c level and hypertension, obesity, or dyslipidemia	Geriatric Depression Scale	Mini Mental State Examination
Iype et al. [33]	2009	71	India	55+	54.9	Cross-sectional	Type 2; doctor diagnosis	Center for Epidemiologic Studies–Depression	Rowland Universal Dementia Assessment Scale
Katon et al. [34]	2010	3 837	USA	18+	47.9	Prospective cohort (5 years)	Types 1, 2; fasting plasma glucose, diabetes medication, doctor diagnosis	Patient Health Questionnaire 9	Incident dementia, International Classification of Diseases-9 codes
McFall et al. [35]	2010	41	Canada	55–81	56.1	Cross-sectional	Type 2; doctor diagnosis, diabetes treatment, onset over age 31	Center for Epidemiologic Studies–Depression	Reaction time
Mejía-Arango et al. [36]	2011	749	Mexico	50+	61.0	Prospective cohort (1–3 years)	Type not specified; self-report and blood test or diabetes treatment	Center for Epidemiologic Studies–Depression	Cross-Cultural Cognitive Examination or Informant Questionnaire on Cognitive Decline in the Elderly
Katon et al. [37]	2012	19 239	USA	30–75	49.0	Prospective cohort (5 years)	Type 2; doctor diagnosis and administrative medical records	Doctor diagnosis and administrative medical records	Incident dementia, administrative medical records
Munshi et al. [38]	2012	145	USA	70–93	52.0	Cross-sectional	Type 2; doctor diagnosis	Geriatric Depression Scale	Dysexecutive Questionnaire
Trento et al. (S3 File) [39]	2012	459	Italy	40–80	47.4	Cross-sectional	Type 2; doctor diagnosis	Zung Self-Rating Depression Scale	Mini Mental State Examination
Koekkoek et al. [40]	2013	366	Netherlands	50–80	43.7	Cross-sectional	Type 2; doctor diagnosis or blood tests	Center for Epidemiologic Studies–Depression or Beck Depression Inventory II	Composite cognition score
Sullivan et al. [41]	2013	2 977	USA, Canada	40–79	46.6	Prospective cohort (40 months)	Type 2; 1997 American Diabetes Association criteria	Patient Health Questionnaire 9	Digit Symbol Substitution Test, Rey Auditory Verbal Learning Test, Stroop Test
Moulton et al. [42]	2015	1 541	UK	18+	44.0	Cross-sectional	Type 2; World Health Organization criteria, determined by physicians	Patient Health Questionnaire 9	Modified Telephone Interview for Cognitive Status

The results collected from each study are shown in Table 3 as originally reported, with no additional adjustment performed by the authors of this systematic review. These results are grouped according to methodological quality of the reported outcome, as determined by the risk of bias assessment reported in Fig 2. The authors and date of publication are stated in the first two columns of the table. The outcome measure being reported is listed in the third column, and adjustment for any critical confounders (i.e., age, ethnicity or race, physical activity, and education) is specified in the fourth column. Depending on the measures of each study, the results are reported in the fifth and/or sixth columns (“Diabetes alone” and “Diabetes and depression”). For analyses that used one of these categories as the reference group for the statistic provided, the reference group is indicated in these columns. Where available, a confidence interval or p-value is presented in the seventh column, with preference given to confidence intervals if both were reported.

**Table 3:** Main results by risk of bias.

Authors	Year	Measure	Adjustment of critical confounders	Diabetes alone	Diabetes and depression	95% confidence interval
**Moderate risk of bias**						
Rotkiewicz et al.	2006	Linear regression coefficient: change in MMSE score	Age, education	[Reference]	-0.03	(-0.05, -0.01)
Katon et al.	2010	Hazard ratio: incident dementia	Age, education, ethnicity, physical activity	[Reference]	2.69	(1.77, 4.07)
Katon et al.	2012	Hazard ratio: incident dementia	Age, education, ethnicity	[Reference]	2.77	(2.48, 3.09)
Sullivan et al.	2013	Difference of means: DSST	Age, education, ethnicity	0.74	[Reference]	(0.27, 1.20)
		Difference of means: RAVLT	Age, education, ethnicity	0.19	[Reference]	(0.08, 0.29)
		Difference of means: Stroop	Age, education, ethnicity	-1.07	[Reference]	(-1.95, -0.20)
**Serious risk of bias**						
Klunder	2005	Linear regression coefficient: change in CVLT score	Age, education	–	-0.23	(-0.56, 0.10)
Umegaki et al.	2008	Logistic regression coefficient: odds of MMSE score ≤ 23	Age	–	1.139	(1.045, 1.243)
Iype et al.	2009	Pearson’s correlation: CESD and RUDAS scores	None	–	-0.36	p = 0.002
Mejía-Arango et al.	2011	Linear regression coefficient: risk of incident dementia	Age, education	2.71	3.78	(1.73, 4.24), (2.37, 6.04)
Munshi et al.	2012	Linear regression coefficient: change in DEX score	Education	–	0.94	(0.31, 1.57)
Trento et al.	2012	Linear regression coefficient: change in MMSE score	Age	–	-0.001	(-0.052, 0.051)
Koekkoek et al.	2013	Mean difference: composite cognition score	Age	[Reference]	0.01	99% CI (-0.15, 0.18)
**Critical risk of bias**						
Bruce et al.	2003	Spearman’s correlation: EBAS-DEP and MMSE scores	None	–	-0.17	p = 0.012
Watari et al.	2006	Difference of means: MMSE scores	None	28.65	28.70	p = 0.91
McFall et al.	2010	Linear regression coefficient: change in reaction time	None	0.15	0.05	p < 0.05
Moulton et al.	2015	Correlation coefficient: PHQ-9 and TICS-M scores	None	–	-0.037	p = 0.15

#### Moderate risk of bias

The four studies with moderate risk of bias found participants with comorbid depression fared worse on cognitive outcomes. These studies presented analyses of prospective cohorts with follow-up time between 40 months and seven years. Combined, they accounted for almost 27,000 participants with diabetes, some of which were older Mexican-Americans, [30] general population samples from the US of a wide range of ages, [34, 37] and participants at high risk of cardiovascular disease from the US and Canada. [41]. One found slightly poorer MMSE scores per each 1-point increase in depressive score, [30] two found significant relative increases in incident dementia for the comorbid group over a five year period, [34, 37] and another found poorer scores in three different cognitive tests for the same group. [41] All of these differences were statistically significant (Table 3).

#### Serious risk of bias

Seven studies were found to have serious risk of bias, some of which provided evidence for poorer cognition measures in participants with comorbid depression and diabetes, while others provided evidence of no association between cognition and comorbid depression and diabetes. Most studies were cross-sectional, save for one prospective cohort study with 3 years of follow-up. [36] Together they accounted for almost 3,000 participants with diabetes, including samples from the US, [29, 38] Japan, [32] India, [33] Mexico, [36] Italy, [39] and the Netherlands. [40] Three of the studies found depressive symptoms were statistically significantly associated with poorer cognitive outcomes. [32, 33, 38] One study reported higher risk of incident dementia in participants with comorbid depression, but estimates were not tested for difference, and their confidence intervals overlapped. [36] Another found increases in depressive scores to be associated with a decrease in verbal learning scores, but the findings were not statistically significant. [29] In contrast, two studies reported estimates that suggest no relationship between depressive symptoms and cognitive scores with considerably narrow confidence intervals (Table 3). [39, 40]

#### Critical risk of bias

Four studies were found to have critical risk of bias, meaning they were likely to be too biased to allow for any contribution to the research question. [26] All four studies were cross-sectional analyses, and in total accounted for less than 2,000 participants with diabetes from Australia, [28] Canada, [35] the UK, [42] and US. [31] There was no clear trend among these studies with respect to an association between depression and cognition. One study found a very weak but statistically significant correlation between depressive symptoms and MMSE scores. [28] Another study found that the two groups’ MMSE means differed by a fraction of a point, with a t-test confirming that these differences were not significantly different. [31] Similarly, another reported no relationship between depressive symptoms and TICS-M scores. [42] Lastly, one study found that reaction times were significantly faster among participants with comorbid depression–the only study out of fifteen to find a positive association between depression and cognition (Table 3). [35]

## Discussion

This systematic search found 15 articles whose data addressed the proposed study question. Quality assessments using the ROBINS-I tool found the risk of bias to be moderate at its lowest, meaning the findings might be considered “sound” despite some problems, [26] and therefore useful to gaining an understanding of the association between depression and cognition in persons with diabetes. The 11 remaining studies were found to have serious or critical risk of bias mainly in the domains of confounding, selection bias, and reporting of results. Information on missing data was often not reported, which precluded a well-informed appraisal of risk in this aspect. Bias due to confounding could have been mitigated by adjusting for adequately measured critical confounders. Bias due to selection and missing data could have been minimized through careful reporting and adjustment methods suited to each case. Finally, bias due to reported results could be avoided by reporting all results of all analyses stated in the paper, regardless of statistical significance. Unfortunately, none of these domains could be adjusted for in this review.

A great deal of heterogeneity was found in the studies with respect to exposure, cognitive outcomes, and participant characteristics. Among the studies with the lowest level of bias alone there are different diagnosis criteria for diabetes and depression, different analyses and measures of cognitive decline, varying ethnic composition of the samples studied, and a range of follow-up times, making it difficult to quantify the relationship between depression and cognition in persons with diabetes. Nevertheless, the broad selection criteria for studies was intended to capture all the studies pertinent to the study question, and this flexibility allowed for a marked trend to be discovered across studies of different characteristics.

In sum, the four least biased results indicated worse cognitive outcomes in participants with elevated depression symptoms relative to those with lower depressive symptom scores. Though all four results came from well-powered studies and indicated statistically significant differences, whether these results translate into clinically important differences in patient populations remains unclear.

### Limitations

There were several limitations to this systematic review. The most salient is the high overall risk of bias in many of the study results. The lowest level of bias observed in these studies was “moderate,” which is often the most favourable level of risk of bias that may be expected among non-randomized studies. [26] Residual confounding is of particular concern, as the exposure of interest—depression—is related to many factors that impact health outcomes and cannot be randomized. As a result, the strength of the evidence found in this review is limited, and the conclusions suggested by the evidence must be tempered by this fact.

This review focused on current depression and depressive symptomatology in order to capture its immediate association with cognitive function. However, it is possible that depression or symptoms occurring throughout the life course may be associated with cognition many years later, especially since depression is highly recurrent. [43] Furthermore, it is possible that the relationship between depression and dementia may vary at different stages of life. For instance, a recent review shows that depression occurring earlier in life is a clear risk factor for dementia later in life, while depression occurring later in life has not yet been adequately shown to be a risk, a prodrome, or an effect of developing dementia. [44] While not the substantive focus of this review, further research may find different patterns of lifetime exposure to depression to have different associations or causal relations with cognitive decline in persons with diabetes.

Despite the lack of formal tests for publication bias, there was a clear bias toward reporting significant results within many of the studies themselves. This most often occurred in analyses where several figures were calculated but only statistically significant estimates were published. This problem may have been exacerbated by the limited statistical power afforded by the small sample sizes used in many of the studies. Several authors were contacted for omitted results, but these efforts were only successful for one study (S3 File).

### Conclusions

Since 1980, the global age-standardized prevalence of diabetes has more than doubled for men and risen by 60% for women, and is projected to continue rising. [45] Diabetes is currently the seventh leading cause of disability worldwide, [46] making its management and complications a high priority for healthcare systems worldwide. In addition, an estimated 47.5 million people have dementia around the world, with the number expected to almost triple by 2050. [47] This review finds compelling evidence that the presence of comorbid elevated depressive symptoms in persons with diabetes is associated with poorer cognitive outcomes than for persons with fewer symptoms. This association was seen in rates of incident dementia, Mini Mental State Examination scores, and tests evaluating various domains of cognitive functioning. At the individual level, healthcare professionals must be aware that in addition to well-established complications of diabetes, persons with diabetes are at high risk of depression, and that people with comorbid depression and diabetes are more likely to suffer from lower cognitive functioning than their peers. Nevertheless, the epidemic levels of diabetes and its complications can only be addressed by preventive public health efforts at societal and global scales.

Further research is needed to quantify the amount of cognitive decline that is attributable to comorbid depression in diabetes, and to confirm if these deficits explain or contribute to clinically important differences in health outcomes. Given the deleterious cognitive outcomes associated with current depression, is important to investigate whether remitted depression is associated with a return to previous cognitive abilities or permanent damage. Research in this area should distinguish between depressive symptoms and clinical depression, as these two conditions may have different associations with subsequent cognitive decline. As stated previously, age at the time of exposure to depression should also be studied for similar reasons. Finally, evaluating the effectiveness of different treatment strategies can identify the most successful methods of supporting illness management in people with comorbid depression and diabetes in order to delay and avoid diabetes complications. Given the inherent difficulties of observational research on depression, each of these proposed paths would require careful reporting of participant selection procedures and missing data, rigorous adjustment for confounding, and full reporting of all analyses stated regardless of statistical significance.

## Supporting Information

S1 File PRISMA Flow diagram. (PDF)

S2 File PRISMA Checklist. (PDF)

S3 File Personal communication with Dr. Marina Trento. (PDF)
