# Supplementary material for: Association between Depressive Symptoms and Cognitive Function in Persons with Diabetes Mellitus: A Systematic Review
Source: PLoS One. 2016 Aug 15;11(8):e0160809. doi: 10.1371/journal.pone.0160809 (PMC4985066; doi:10.1371/journal.pone.0160809)
Supplement: S1 File — (PDF) [file pone.0160809.s001.pdf]

PRISMA 2009 Flow Diagram

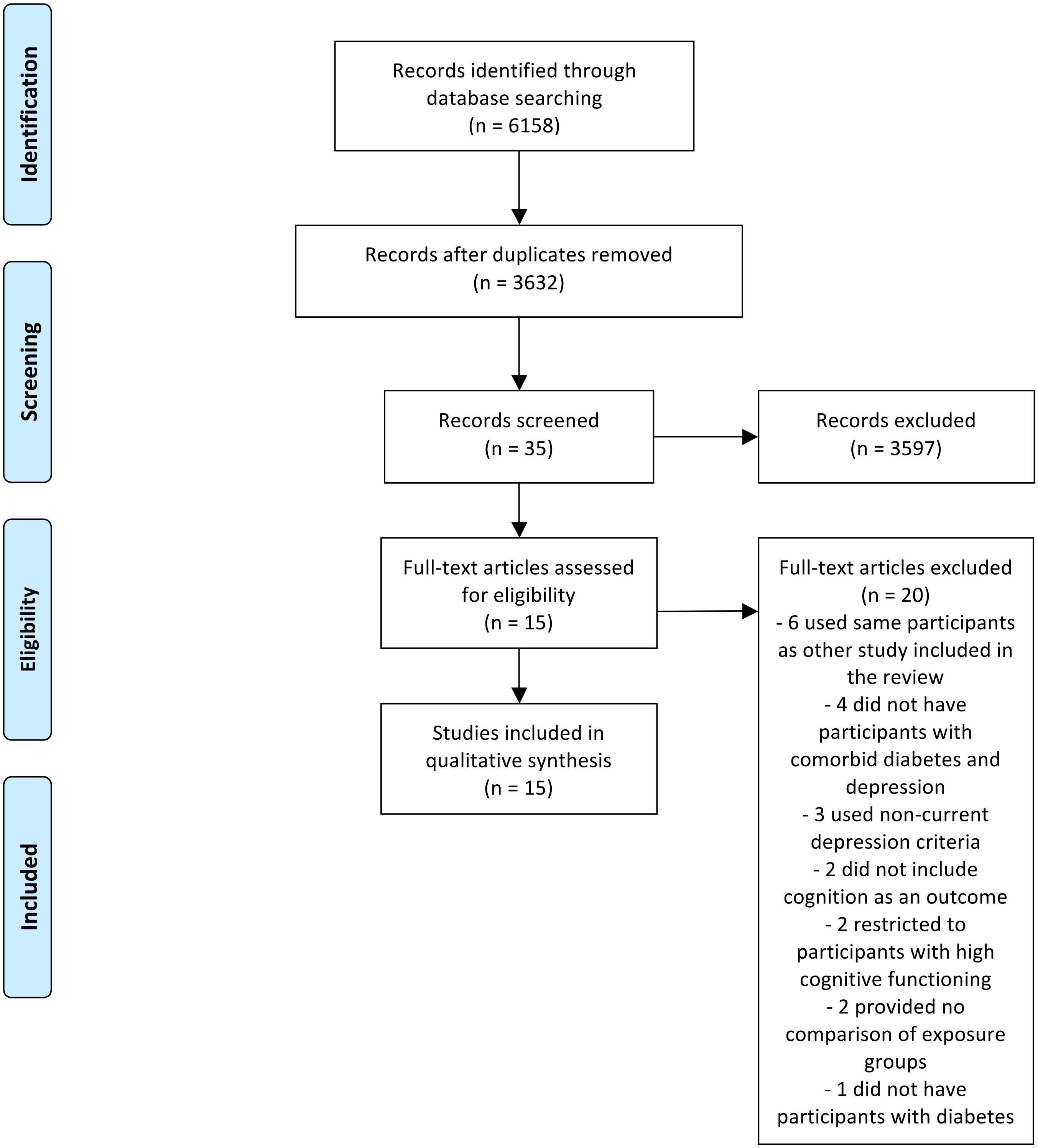

From: Moher D, Liberati A, Tetzlaff J, Altman DG, The PRISMA Group (2009). *P*referred *R*eporting *I*tems for *S*ystematic Reviews and *M*eta-*A*nalyses: The PRISMA Statement. PLoS Med 6(7): e1000097. doi:10.1371/journal.pmed1000097

For more information, visit www.prisma-statement.org.
