# Supplementary material for: Association between Depressive Symptoms and Cognitive Function in Persons with Diabetes Mellitus: A Systematic Review
Source: PLoS One. 2016 Aug 15;11(8):e0160809. doi: 10.1371/journal.pone.0160809 (PMC4985066; doi:10.1371/journal.pone.0160809)
Supplement: S3 File — (PDF) [file pone.0160809.s003.pdf]

Re: Question about your 2012 article

Sofia Danna

Fri 2016-02-19 12:03 PM

To:Marina Trento <marina.trento@unito.it>;

Cc:Lorena Charrier <lorena.charrier@unito.it>; Franco Cavallo <Franco.cavallo@unito.it>;

Categories: Systematic review

Dear Dr. Trento,

Thank you so much for your prompt response, this was exactly what we needed.

All the best,
Sofia

From: Marina Trento <marina.trento@unito.it>
Sent: February 19, 2016 4:42 AM
To: Sofia Danna
Cc: Lorena Charrier; Franco Cavallo
Subject: Re: Question about your 2012 article

Dear Sofia,

thanks for your message. To answer your question I consulted our statisticians, who read a copy.
Attached is the table with the data you requested.
We remain at disposal for others questions.

Best regards,
Marina Trento

Il 18/02/2016 18:14, Sofia Danna ha scritto:

> Dear Dr. Trento,
>
> I am a graduate student in Epidemiology currently working on a systematic review on cognition in people with diabetes and depression (see our protocol for more information). We have included your 2012 article entitled "A cross-sectional survey of depression, anxiety, and cognitive function in patients with type 2 diabetes" in our final screening, along with 22 other articles. In reporting the results of the third multivariate model you state that "None of the clinical independent variables correlated with MMSE" but do not state any specific coefficients or p-values. In order to compare these results with those of the other papers we are including, it would be helpful to have the exact coefficients and p-values for this regression analysis. Might you be able to send us those estimates?

Thank you very much in advance,

Sofia M. Danna
M.Sc. Epidemiology Candidate
McGill University
sofia.danna@mail.mcgill.ca

--
Dr. Marina Trento
Pedagogist. MBA
Laboratory of Clinical Pedagogy
Department of Medical Sciences
University of Turin. C.so Dogliotti, 14
10126 Torino
FAX 0039 011 6708436
Torino, 10126, Italy

MMSE	Coef.	Std. Err.	t	P>|t|	[95% Conf.	Interval]
ZUNG_Anx	-.0501908	.0269168	-1.86	0.063	-.1030896	.002708
ZUNG_Dep	-.0008058	.0261676	-0.03	0.975	-.0522323	.0506207
age	.0258577	.0211757	1.22	0.223	-.0157583	.0674736
diab_duration	-.0198837	.0207172	-0.96	0.338	-.0605987	.0208313
Hba1c	-.036296	.1116092	-0.33	0.745	-.2556387	.1830467
Group_care	.2393341	.425043	0.56	0.574	-.5959916	1.07466
sex	.1478704	.3312421	0.45	0.656	-.5031109	.7988517
trp_new*	-.3653567	.3429484	-1.07	0.287	-1.039344	.3086306

*IT (insulin treatment) vs NIT (non insulin treatment)
